# Development and testing of gold nanoparticles for drug delivery and treatment of heart failure: a theranostic potential for PPP cardiology

**DOI:** 10.1186/1878-5085-4-20

**Published:** 2013-07-29

**Authors:** Mykola Ya Spivak, Rostyslav V Bubnov, Ilya M Yemets, Liudmyla M Lazarenko, Natalia O Tymoshok, Zoia R Ulberg

**Affiliations:** 1 Zabolotny Institute of Microbiology and Virology, National Academy of Sciences of Ukraine, Zabolotny str., 154, Kyiv 03680, Ukraine; 2 LCL “DIAPROF”, Svitlycky str., 35, Kyiv 04123, Ukraine; 3 Centre of Ultrasound Diagnostics and Interventional Sonography, Clinical Hospital “Pheophania” of State Affairs Department, Zabolotny str., 21, Kyiv 03680, Ukraine; 4 Scientific-Practical Centre of Pediatric Cardiology and Cardiac Health of Ukraine, Chornovil str., 28/1, Kyiv 01135, Ukraine; 5 Ovcharenko Institute of Biocolloidal Chemistry, National Academy of Sciences of Ukraine, Acad. Vernadsky blvd, 42, Kyiv 03142, Ukraine

**Keywords:** Predictive, Preventive, Personalized medicine, Nanomedicine, Gold nanoparticles, Heart failure, Drug delivery, Sonoporation, Theranostics, Animal model

## Abstract

**Introduction:**

Nanoscale gold particles (AuNPs) have wide perspectives for biomedical applications because of their unique biological properties, as antioxidative activity and potentials for drug delivery.

**Aims and objectives:**

The aim was to test effects of AuNPs using suggested heart failure rat model to compare with proved medication Simdax, to test gold nanoparticle for drug delivery, and to test sonoporation effect to increase nanoparticles delivery into myocardial cells.

**Material and methods:**

We performed biosafety and biocompatibility tests for AuNPs and conjugate with Simdax. For *in vivo* tests, we included Wistar rats weighing 180–200 g (*n* = 54), received doxorubicin in cumulative dose of 12.0 mg/kg to model advance heart failure, registered by ultrasonography. We formed six groups: the first three groups of animals received, respectively, 0.06 ml Simdax, AuNPs, and conjugate (AuNPs-Simdax), intrapleurally, and the second three received them intravenously. The seventh group was control (saline). We performed dynamic assessment of heart failure regression *in vivo* measuring hydrothorax. Sonoporation of gold nanoparticles to cardiomyocytes was tested.

**Results:**

We designed and constructed colloidal, spherical gold nanoparticles, AuNPs-Simdax conjugate, both founded biosafety (in cytotoxicity, genotoxicity, and immunoreactivity). In all animals of the six groups after the third day post-medication injection, no ascites and liver enlargement were registered (*P* < 0.001 vs controls). Conjugate injection showed significantly higher hydrothorax reduction than Simdax injection only (*P* < 0.01); gold nanoparticle injection showed significantly higher results than Simdax injection (*P* < 0.05). AuNPs and conjugate showed no significant difference for rat recovery. Difference in rat life continuity was significant between Simdax vs AuNPs (*P* < 0.05) and Simdax vs conjugate (*P* < 0.05). Sonoporation enhances AuNP transfer into the cell and mitochondria that were highly localized, superior to controls (*P* < 0.01 for both).

**Conclusions:**

Gold nanoparticles of 30 nm and its AuNPs-Simdax conjugate gave positive results in biosafety and biocompatibility *in vitro* and *in vivo*. AuNPs-Simdax and AuNPs have similar significant cardioprotective effects in rats with doxorubicin-induced heart failure, higher than that of Simdax. Intrapleural (local) delivery is preferred over intravenous (systemic) delivery according to all tested parameters. Sonoporation is able to enhance gold nanoparticle delivery to myocardial cells *in vivo*.

## Overview

### Predictive, preventive, and personalized cardiovascular nanomedicine

In the developing paradigm of predictive, preventive, and personalized nanomedicine (PPPM), a crucial point is highly specific and sensitive, i.e., drug targeting, to make patients receive the right drug for their disease at the right dose and the right time [1]. One of the goals of PPPM is to diagnose, observe the process of treatment in tissue transformation, and analyze early parameters (biomarkers) to assess/predict the outcome, driven by a decision-making process.

Heart diseases are one of the main causes of death worldwide; heart failure is associated with a significantly reduced physical and mental health, resulting in a decreased quality of life [2,3]. Although many patients with cardiovascular diseases survive for many years, the progressive disease is associated with an overall annual mortality rate of 10% [4]; heart failure is the leading cause of hospitalization in people older than 65 years [4].

Some of the outstanding achievements at the end of the last century are the studies on properties of biological and synthetic materials in nanometer size. The rapid development of nanoscience has caused the formation of fundamentally new directions for biotechnology research on nano-objects, which are characterized by peculiar, often unexpected properties that are different from the properties of both macro- and microscale particles.

Development of fundamentally new methods of diagnosis and treatment goes towards the use of nanomaterials (NPs) and nanotechnology, which has a very broad definition based on scale, and nanomedicines are likewise based not only on the type of medicine or their function but also on a nanosized range. While most nanotechnology is expected to have an upper size limit of 100 nm, in the drug delivery field, this is more generally accepted as medicines in the size range from a few nanometres to 1,000 nm in diameter. Advances in nanotechnology have led to the development of new materials and devices for various scientific and therapeutic purposes.

### Modeling of heart failure

Mouse and rat models are widely used in biological and medical sciences, but these studies are still limited by the inability of noninvasive collection of anatomical and physiological data during the time of the study. The use of rats as animal models is rational from the economic viewpoint, and many techniques have been developed to measure relevant functional parameters. Animals have been used by humans to understand their own biology since many human diseases are studied on experimental animals. The appropriateness of animal models in the future is determined by the fact that even the most powerful computers using appropriate mathematical models are unable to reproduce the interaction between molecules of cells, organs, organisms, and environment [5].

In cardiovascular research, animal models have allowed the study of cardiovascular disease in the early stages, as well as the investigation on the mechanisms of the pathogenesis of cardiovascular disease and the effects of drug intervention. Doggrell et al. [6] suggest that an ideal animal model for any cardiovascular disease in humans should follow five characteristics: (1) mimic the human disease, (2) allow studies in chronic, stable disease, (3) produce symptoms which are predictable and controllable, (4) satisfy economical, technical, and animal welfare considerations, and (5) allow measurement of relevant cardiac, biochemical, and hemodynamic parameters. Cardiovascular disease is uncommon in young humans but markedly increases with age [7].

The numeral rat models of hypertension, cardiac hypertrophy, and heart failure were suggested for the following conditions [8]: hypertension, systemic spontaneously hypertensive rats (SHRs), stroke-prone SHR (SHR-SP), mineralocorticoids (DOCA–salt), NO synthase inhibition (L-NAME administration), transgenics (TGR(mREN2) 27 rats), diabetic hypertensive rats (STZ-SHR, Zucker), renal renal artery occlusion (1K1C, 2K1C), pulmonary monocrotaline, hypoxia (normobaric, hypobaric), hypertrophy, spontaneously hypertensive rats (SHRs), renal artery occlusion; pressure loading (aortic banding), catecholamines (noradrenaline, isoprenaline), Transgenics, heart failure, systemic hypertension spontaneously hypertensive rats (SHRs-F), hypertensive HF prone rats, Dahl/Rapp salt-sensitive rats, ischemic heart failure non-occlusive coronary ligation, myocardial infarction coronary ligation, microembolization of coronary vessels, cardiomyopathy toxins (adriamycin, ethanol), myocarditis autoimmune, Chagas' disease, pulmonary hypertension, monocrotaline, hypoxia, and miscellaneous aortacaval fistula (shunts).

### Imaging for heart failure assessment in rats

In the biological sciences, many studies using mouse models are limited by the inability to gather anatomical and physiological information noninvasively in a longitudinal manner. To investigate the changes, the experimental animal had to be killed and dissected. Therefore, reducing the losses and increasing the number of small animals as an experiment by intravital evaluation of changes in animal tissue is a very relevant task. Clinical ultrasound (US) diagnostics besides the human could be successfully used to study the structure of animal tissues, including small ones. The US method is used for research purposes. Ultrasound biomicroscopy (UBM) may overcome this obstacle as it confers near-microscopic resolution through the use of high-frequency ultrasound waves, which can be applied for echocardiography for a fetal study of the cardiovascular system in mouse embryos [9].

Still, preclinical imaging techniques in small animals, especially with the use of ultrasound technology, are used only in few research centers using special equipment around the world [10]. Recently, we performed a study describing the use of US equipment of general use for *in vivo* study of novel medicine testing in mice [11,12] and patented the method of experiment on animals using US [8,13], which consider registration of dynamic changes in the tissues of animals carried ultrasound small laboratory animals (rats, mice) using high-frequency probes using Doppler, sonoelastography, contrast media, motion detection of tissue, interventions under US guidance for administration of drugs, and obtaining material for study and create vector three-dimensional scenes using the received ultrasonic data.

The application of ultrasonic methods using special equipment for *in vivo* examination of cardiac function was also described in rats with echocardiography [14-24]. Bjornerheim et al. [14], while evaluating echocardiography data, considered that Doppler data are more representative than the M-mode. The regional heart function is reported to be exactly evaluated using tissue Doppler and two-dimensional (2D) strain echocardiography [15]. Color Doppler-guided evaluation of aortic flow and aortic root measurement was reported for assessment of stroke volume and cardiac output in mice [16]. Recently, reports have emerged regarding the use of intravascular probes adapted for transesophageal study of the rat heart [15].

Few researches focus on the study of echocardiographic changes in post-infarction of rats with congestive heart failure (CHF) and spontaneous hypertension [18,19,25]. Sjaastad et al. [21] evaluated the post-infarction myocardial function of rats and determined echocardiographic criteria for heart failure CHF using high-performance echocardiography. Extensive myocardial infarction (MI) was induced in rats by left coronary occlusion. Sham-operated animals served as controls. Authors conclude that by high-frame-rate echocardiography, it is possible to obtain high-quality recordings in rats. It is feasible to distinguish MI rats from CHF rats due to the myocardial dysfunction from those without failure and since longitudinal studies are performed on myocardial function. Kokubo et al. [19] presented a study on hypertension, designed to provide a noninvasive evaluation of the time-dependent alteration of cardiac function in male spontaneously hypertensive rat at 4 to 24 weeks of age and age-matched Wistar-Kyoto rats. Echocardiographic studies were performed after blood pressure and heart rate were measured by a tail-cuff method. Rats are commonly used to study left ventricular (LV) hypertrophy and measure the LV mass and dimensions. De Simone et al. [22] determined the accuracy of echocardiography in rats. Blinded cross-sectional area and LV mass measurements using either the cube function or an elliptical model from high-resolution M-mode echocardiograms were compared to the necropsy LV weight (0.28 to 1.5 g) in 41 normotensive (body weight 116 to 762 g) and 17 hypertensive rats (350 to 560 g), comparing postmortem chamber volumes in 28 normal rats (0.02 to 0.19 ml) to echocardiographic volumes derived from the elliptical model. Echocardiography can be used to evaluate LV structure and function in rats and to detect *in vivo* LV anatomic differences induced by hypertension.

In studies of CHF treatment, it is essential to select animals with a similar degree of cardiac dysfunction. However, this is difficult to establish without hemodynamic evaluation in rat post-infarction-induced congestive heart failure. Martinez et al. [23] studied the diagnostics of congestive heart failure in long-term follow-up in post-infarction rats using only echocardiographic criteria through a J-tree cluster analysis and Fisher's linear discriminant function in two sets of sham and infarcted rats. Echocardiographic analysis has shown to be useful in accurately predicting congestive heart failure in postinfarction rats with 100% specificity and 80% sensitivity. However, ultrasound can cause complication in rats, such as pulmonary hemorrhage [24].

### Doxorubicin for heart failure modeling

*Doxorubicin*, one of the most effective anticancer drugs, is characterized by severe cardiotoxic effects, which induce cardiac remodeling and congestive heart failure. The method of simulation of heart failure uses doxorubicin [26] by means of 10 intraperitoneal drug injection in dose of 1 mg/kg, followed by transesophageal echocardiography adapted intravascular probe. Kharin et al. [27] described chronic doxorubicin cardiotoxicity in rats in a cumulative dose of doxorubicin (15 mg/kg) by six equal intraperitoneal injections in a 2-week period. Authors registered remodeling of ventricular repolarization heterogeneity. The major findings were as follows: (1) activation-recovery intervals (ARIs) on the ventricular epicardium of both ventricles were significantly prolonged in the doxorubicin group and (2) this inhomogeneous prolongation of ARIs on the ventricular epicardium resulted in the increase in the dispersion of repolarization across the ventricular epicardium and the inhomogeneous alterations of the regional ARI gradients on the ventricular epicardium. These changes in repolarization could explain the electrocardiographic alterations, that is, the prolongation of the QT interval and flattening of the T wave.

In clinical practice, despite its cardiotoxic effects, doxorubicin remains in use because of its efficacy in the treatment of several types of tumors [28]. According to Steinherz et al. [29], echocardiography in humans should be performed before every additional course of doxorubicin up to a total dose of 300 mg per square meter, given with or without concurrent radiation therapy. A change in the left ventricular ejection fraction, as determined by echocardiography, is a very good indicator of developing cardiomyopathy; monitoring for such a change should be frequent during treatment and regular thereafter throughout the patient's lifetime. Adjunctive therapy with an antioxidant such as probucol merits serious consideration. Endomyocardial biopsy remains the most sensitive method for early diagnosis of ensuing cardiomyopathy.

Dimitrakis et al. [30] tested the hypothesis of the dose-dependent cardiotoxicity of doxorubicin, affecting protein degradation pathways in adult cardiomyocytes connected to the effects of apoptosis, autophagy, and the proteasome/ubiquitin system in long-term-cultured adult rat cardiomyocytes. Thus, doxorubicin causes a down regulation of the protein degradation machinery of cardiomyocytes with a resulting accumulation of poly-ubiquitinated proteins and autophagosomes. Although autophagy is initially stimulated as a compensatory response to cytotoxic stress, it is followed by apoptosis and necrosis at higher doses and longer exposure times. This mechanism might contribute to the late cardiotoxicity of anthracyclines by accelerated aging of the postmitotic adult cardiomyocytes and to the susceptibility of the aging heart to anthracycline cancer therapy.

Al-Shabanah et al. [31] studied interaction of doxorubicin with iron and the consequent generation of reactive oxygen species as a major player in doxorubicin-induced cardiomyopathy.

It was supposed that the pericardial pores may function in an allied self-defense mechanism between the pleural and pericardial cavities in mice [32]. Numerous circular fenestrations or pores that indirectly connect the right and left pleural cavities were present in the pericardium of the golden hamster and rat [33]. This phenomena could be relevant in choosing the place of injection (the effect should be expected as similar).

However, in described models, focus mostly on local heart function assessed by echocardiography, the dynamic *in vivo* examination of systemic circulation of the animals was not sufficiently evaluated. All cited papers using ultrasound imaging, considered application of special US equipment.

We did not find any data regarding the use of precise injection under US guidance for heart failure modeling in rats. Without visual navigation, injection methods are still limited by introduction of agents orally, into the tail vein, intraperitoneally, and subtentorially. Finally, developing a comprehensive methodology for the cardiovascular system using Doppler, M-mode parameters, conduction systematic assessment of hemocirculation, injection drugs under US guidance in pericardial and pleural cavities remains to be an important task; optimal cardiotoxic dose of doxorubicin for extended and longitudinal observation of rats has not been determined.

Recently, we described and patented the method of doxorubicin-induced heart failure on the rat model using the US equipment, which focuses on peripheral circulation. The assessment suggested that optimal cardiotoxic dose of doxorubicin for extended and longitudinal observation of rats has not been determined [10,34]. The modeling process for heart failure, which includes an experiment on laboratory animals (rats) introduced with a cardiotoxic drug (doxorubicin) and assessed *in vivo* using a dynamic ultrasound, can be used for research purposes, i.e., for basic preclinical studies of new drugs, and can be recommended for implementation purposes to research institutes, centers, departments of cardiology, ultrasound, and interventional ultrasonography. US is an effective modality for *in vivo* monitoring of the condition of rat organs targeted for experiment in the study of cardiovascular function. In this study, the optimal dose of doxorubicin was established. Thus, the dose higher than 23.1 mg/kg leads to the death of animals from the 20-day experiment, while the dose lower than 12.45 mg/kg did not induce significant clinical symptoms. US criteria to gain reproducible model and cardiovascular symptoms are compensated to the level of normal parameters in a short period. Four- and five-time administrations of doxorubicin showed similar results for modeling heart failure.

The experimental rat organs were investigated by authorities in similar criteria and patterns as ultrasound diagnostics are performed in humans. We obtained average ultrasonic linear parameters of the rat organs: (before receiving doxorubicin) the longitudinal liver size 15 ± 1.5 mm, longitudinal kidney size 16 ± 1.3 mm, longitudinal size of spleen 14 ± 1.7 mm, inferior vena cava diameter was 3.0 ± 2,3 mm, diameter of portal vein 1.5 ± 0.3 mm, ejection fraction 78% ± 6%, maximum systolic velocity in aortic root was 11 ± 1.3 cm/s, resistive index (RI) in the renal segmental arteries 0.68 ± 0.04. Sizes were compared with necropsies. These average linear parameters of the main organs of normal rats and rats with heart failure, which received a cumulative dose of doxorubicin (15 mg/kg; Sigma-Aldrich, St. Louis, MO, USA) had significant changes in mentioned organs (Table 1).

**Table 1:** **Sonometry data of rats**[34]

**Parameter**	**Control group**	**Model of heart failure**	***P***
Longitudinal liver size (mm)	15.0 ± 1.5	18.0 ± 1.7	<0.05
Longitudinal kidney size (mm)	16.0 ± 1.3	17.0 ± 1.4	>0.05
longitudinal spleen size (mm)	14.0 ± 1.7	17.0 ± 1.8	<0.05
Inferior vena cava, diameter (mm)	3.0 ± 2.3	3.7 ± 2.6	>0.05
Portal vein, diameter (mm)	1.5 ± 0.3	1.78 ± 0.2	<0.05
Ejection fraction (%)	78.0 ± 6.0	46.0 ± 8.0	<0.01
Maximum systolic velocity aortic root (cm/sec)	11.0 ± 1.3	6.5 ± 1.6	<0.01
Resistive index (RI) in the renal segmental arteries	0.68 ± 0.04	0.7 ± 0.04	>0.05

We concluded that US is an effective modality for *in vivo* monitoring of rat organs targeted in experiments for the study of cardiovascular function. Interventional ultrasonography is effective for expanding of the utility of modeling and drug testing. However, the method is operator related and requires US specialized training, particularly in small animals. A suggested model (The optimal dose of doxorubicin for simulation of CHF of 2.5 mg/animal is a cumulative dose of 12.45 mg/kg in four injections every 3 days) can be used for research purposes and basic or preclinical studies of new drugs and can be recommended for implementation purposes in research institutes.

*Interventional experiments* on rats showed [34] that the technique is feasible for an expanding experiment: injection into the pericardial and the pleural rat cavities showed a non-specified difference between the two approaches.

### Simdax (levosimendan)

For comparison, we chose the calcium sensitizer *levosimendan* (Simdax) as the most effective inotropic agent that improves myocardial contractility in patients with heart failure; although, its effects on inflammation and apoptosis are unknown. Trikas [35] examined the effects of levosimendan on markers of inflammation and apoptosis, over a period of 30 days following a 24-h infusion, in patients with heart failure. Their findings indicated that levosimendan decreases the expression of proinflammatory cytokines, tumor necrosis factor (TNF)-α receptors and sFAS, immediately after infusion, an effect which persists for 7–30 days.

### The clinical application of gold nanoparticles

Plasmonic (noble metal) nanoparticles distinguish themselves from other nanoplatforms such as semiconductor quantum dots, magnetic and polymeric nanoparticle by their unique surface plasmon resonance [36-42]. Nanogold (also called gold nanoparticle or colloidal gold) has been actively investigated in a wide variety of biomedical applications due to its biocompatibility and ease of conjugation to biomolecules [43,44] and thus offering multiple modalities for biological and medical applications [45]. The non-cytotoxicity, non-immunogenicity and biocompatibility of many AuNPs make us relatively optimistic concerning their future essential applications in nanomedicine [43,46].

### Diagnostics

Gold nanoparticles are also used to detect biomarkers in the diagnosis of heart diseases, cancers, infectious agents (e.g., home pregnancy test) [46], and Alzheimer's disease [47,48]. Multivalent AuNPs were found to inhibit HIV fusion [49], and AuNPs-hepatitis B virus (HBV) DNA was successfully prepared and could potentially apply to multi-gene detection chips [50]. A successful application of the AuNP-nanoprobe was the sensitive detection in clinical samples of *Mycobacterium tuberculosis*[51]. Diabetes was characterized as a multifactorial disease using the AuNP-nanoprobe method mentioned above and involving the capture of the analyte with a magnetic particle featuring recognition elements followed by binding of a AuNP with a second-recognition agent and marker DNA strands for cancer detection [52].

### Therapeutic agent delivery

The study on drug delivery by nanoparticles is highly perspective of personalized medicine in the future [53]. Over the past decade, several delivery vehicles have been designed based on different nanomaterials, such as polymers [54], dendrimers [55], liposomes [56], nanotubes [57], and nanorods [58]. Therapeutic agents can also be coated onto the surface of gold nanoparticles. The large surface area-to-volume ratio of gold nanoparticles enables their surface to be coated with hundreds of molecules (including therapeutics, targeting agents, and anti-fouling polymers) [59].

Therapeutic vectors carry drugs, genes, and imaging agents into living cells and tissues [38,60]. The drug vectors should also be stable in the circulatory system yet can also become labile under appropriate conditions when the targeted organ is reached. The drug vectors carry the drug by encapsulation or more or less strong binding (covalent, coordination, or supramolecular bond) [61]. Different routes of administration can result in various effects on the biodistribution of drug carriers.

### Sonoporation

Vibration caused by ultrasonic waves can change the structure of the cell membrane and enhance its permeation. A new ultrasound-aided method, sonoporation, has been proposed and utilized to transmit target molecules (such as drugs and DNA) into cells for therapy [61]. Yu Hsin et al. [62] showed approximately 60% improvement in terms of fluorescence signals from the cellular uptake of gold nanoparticles after sonoporation treatment. Therefore, we conclude that the controlled release is feasible and can further improve the therapeutic effects of the nanoparticles [63]. Our recent results demonstrated that polyplex gene transfer by US exposure is effective. It illustrated the potential of ultrasound-triggered gene delivery technology for gene therapy [64]. Therefore, we conclude that the controlled release is feasible and can further improve the therapeutic effects of the nanoparticles.

### Treatment

Gold nanoparticles are being investigated as carriers for drugs [36]. Kogan et al. utilized AuNPs in weak microwave fields in order to dissolve amyloid aggregates [37]. It was reported that gold nanoparticles were utilized for diagnostics [38] and cancer treatment [39].

The application of nanoparticles allows the combination of therapy and diagnosis, known as *theranostics*, which has received increasing attention in biomedicine [65].

### Bioeffects of AuNPs

Oxidative stress is one of the main factors in cellular aging and other cellular disorders [66]. While therapeutic treatments cannot be based exclusively on the abatement of the oxidative stress, a neutralization of this cellular disorder could minimize collateral damages associated to the transformation of biomolecules in the cytosol. Traditionally, reactive oxygen intermediates were considered to be toxic by-products of aerobic metabolism, which were disposed of using antioxidants. Superoxide radicals and hydrogen peroxide [67] balance, together with the sequestering of metal ions, is thought to be important to prevent the formation of the highly toxic hydroxyl radical via the metal-dependent Haber–Weiss or the Fenton reactions.

Gold nanoparticles have been showing an antioxidant effect in a model of diabetes [68]. The use of Au/CeO_2_ composites allows a large extent from the ability of gold to trap carbon-centered radicals as well as to decompose hydroperoxides [69,70] and also has a strong antioxidant activity against cellular oxidative stress.

### Assessment of risk of nanomaterials

The great expectations of gold colloid use for therapeutic purposes suggests that AuNPs should be biocompatible [43]. There is considerable potential use of AuNPs in nanomedicine, especially for imaging, diagnostics, and therapy, however, toxicity needs to be thoroughly examined with maximum care and accuracy. Because of their surface properties and very small size, nanotubes may bind and transport toxic chemical compounds as well as being toxic themselves by generating free radicals [71], inducing oxidative stress, and thus becomes a disadvantage for their application in medicine [35]. Seaton et al. established potential factors of toxicity of nanoparticles [71], which include length (greater than 15 μm, below it the fiber can be removed by pulmonary macrophages), diameter (less than 3 μm, allows fibers to be inhaled into the gas-exchanging part of the lung), insolubility, resistance to dissolution in the lung environment, and sufficient dose of delivery to the target organ. Particular attention in the context of medical use should be paid to the toxicity nanogold.

A number of studies indicate that AuNPs have low *cytotoxicity* and high *biocompatibility*[36,72-76]. Despite this lack of research on the nanogold toxicity in vivo, a necessary step is needed before the clinical re-dwelling drugs from AuNPs [74,75]. The cytotoxicity of AuNPs, i.e., their cellular toxicity, has been examined by our research groups [36,73,74]. Since everything is toxic at high dose, the important question is whether AuNPs are toxic at the concentration at which they will be used (believed to be in the range of 1–100 AuNPs per cell). Also, *in vivo* conditions are different from *in vitro* results, and in particular, more *in vivo* studies are called for. Thus, no general conclusion can be drawn at present. It has been suggested, however, that it could be applicable to use AuNPs as reference nanoparticles for low toxicity in the set-up of a nanoparticle toxicity scale, given the higher toxicity of carbon nanotubes and quantum dots compared to non-cationic AuNPs. Finally, AuNPs are redox active and reduce the production of reactive oxygen and nitrite species [75].

Thus to the toxicity survey, it appears that AuNPs usually show rather little toxicity, if any, because many cytotoxicity studies report negative cytotoxicity results.

Cardioprotective properties of gold nanoparticles, particularly for heart failure as well as in drug delivery, were still not conclusively confirmed that a call for study using a reliable model is needed.

We suggest to increase the clinical efficacy of treatment to patients with heart failure using nanoconstructions based on gold nanoparticles due to their promising biological properties, as well as antioxidative activity and potentials for drug delivery.

*The aim* of the study is to develop new evidence-based approaches in the synthesis of biologically safe and biocompatible gold nanoparticles and gold-based nanoconstructions with cardiotropic drugs to improve their delivery addressed to cardiovascular pathologies.

*The purposes* of the study:

1. to assess biosafety, biocompatibility, and biological effectiveness of gold nanoparticles and AuNPs-Simdax conjugate created from gold nanoparticles and cardiotropic drug Simdax,

2. to conduct comparative preclinical testing of proved medication Simdax, gold nanoparticles, and AuNPs-Simdax nanoconstruction on suggested heart failure rat model, and

3. to test the sonoporation effect to increase nanoparticle delivery into myocardial cell.

## Methods

### The first stage: biosafety and biocompatibility tests of gold nanoparticles and conjugate with Simdax [36,60,73,74]

We developed and implemented original protocols for colloid-chemical synthesis of spherical gold nanoparticles of discrete sizes 10, 20, 30, and 45 nm; developed, designed, and optimized conjugate based on the cardiotropic agent Simdax and AuNP size of 30 nm, (AuNPs-Simdax). Conjugation was performed at component ratio 1:1 by volume. The final concentration of the active substance conjugate were Simdax 1.25 mg/ml, AuNPs of 30 nm, 19.3 mg/ml by metal. Gold nanoparticles were obtained by chemical condensation, by restoring a gold-hydrochloric acid, sodium citrate in the presence of potassium carbonate. The working solution of gold nanoparticles of size 30 nm were prepared using a 5% glucose solution so that the concentration of gold nanoparticles in solution was 1.17 mg/kg.

We received the colloid gold nanoparticles according to the following reactions:

(1) $$\begin{array}{c} 2\mathrm{HAuC}l_{4}+5K_{2}CO_{3}\to 2\mathrm{KAu}O_{2}+5CO_{2}+8\mathrm{KCl}\\ \;+H_{2}O \end{array}$$

(2) $$\begin{array}{c} 2\mathrm{KAu}O_{2}+2СH_{3}\mathrm{СOС}H_{3}+K_{2}CO_{3}\to 2Au^{0}\\ \;+3СH_{3}\mathrm{СOOK}+\mathrm{KНC}O_{3}+{Н}_{2} \end{array}$$

The structure of gold nanoparticles synthesized by reaction (1) may be represented by the formula:

(3) $${\left\{m\left[Au^{0}\right]n\mathrm{Au}O_{2}{}^{-}:\left(n‐x\right)K^{+}\right\}}^{-x}xK^{+},$$

where *m* is the number of molecules Au^0^ and *n* is the number of excess ions AuO_2_^-^ firmly absorbed on the surface of the unit (usually *m* >*n*) which are potential forming. *x* is the number of ions within the diffusion layer, and (*n*-*x*) is the number of counterions К^+^ absorbed at the layer. The number of potassium ions (*n*-*x*) less than the number of absorbed ions AuO_2_^-^ (*n*) results in the nanoparticle having a negative charge (*s*). The method used to obtain gold nanoparticles allows stable aqueous dispersions of nanoparticles of a certain size to be obtained.

The method used allows to obtain gold nanoparticles and stable aqueous dispersions of nanoparticles of a certain size.

Tests for biosafety and biocompatibility of gold nanoparticles and conjugate were performed according to standard requirements, assessing the appearance (color, size, and shape) of nanoparticles, contamination by extraneous bacterial and fungal microflora, and mycoplasma contamination. Biosafety *in vitro* was performed: genotoxicity in terms of H^+^-ATP-ase activity, Na^+^, K^+^-ATP-ase activity, lactatedehydrohenase activity, mutagenicity, impact on the gastrointestinal tract normal flora, biosafety *in vivo* in terms of genotoxicity test, micronucleus test, immunotoxicity, harmlessness *in vivo* in injecting white mice, and stability in model systems of blood.

For *in vitro* experiments, U937 (human leukemic monocyte lymphoma) cell line has been used. The AuNPs DNA-damaging activity *in vivo* has been estimated by the ‘comet assay’ method (alkaline gel electrophoresis of isolated eukaryotic cells). The working solution was injected into the tail vein of rats, and after 1 h, conducted the sampling of material for research.

We performed complex studies aimed at assessing the nature of the influence of gold nanoparticles on Ca^2+^, Mg^2+^-ATP-ase activity in myofibrils. Myofibrils from the cardiac muscles were isolated according to the Solaro method modifications. The concentrations of active ingredients in conjugate were calculated based on the therapeutic dose of Simdax and biosafety concentration of AuNPs, in which they observed minimal inhibitory effect on the amount of Ca^2+^, Mg^2+^-ATP-ase activity in myofibrils.

### Assessment of cardioprotective properties and route of delivery of gold nanoparticles

We used 48 2-month-old laboratory Wistar rats weighing 180–200 g (*n* = 54) of each sex selected on the basis of analogies for the second stage and four rats for the third stage of experiment. Within 2 weeks after intravenous injection of doxorubicin Sigma solution in a cumulative dose of 12.0 mg/kg to simulate heart failure according to experimentally established scheme [34], animals showed an advanced heart failure, registered by US.

**Figure 1:**
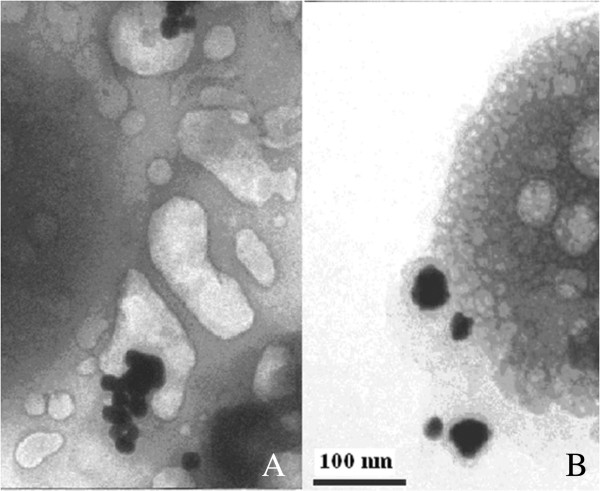
General view if AuNPs of discrete sizes 10, 20, 30, and 45 nm.

For US scanning, 3–12-MHz frequency for linear and microconvex transducers were used. The rats were examined while sedated in a supine position (Figure 1), with the chest closed and the transducer placed gently in the left parasternal position for echocardiography. Multi-planar approaches were utilized for abdominal and pleural cavity examination. Soft fixation of animals was provided. General anesthesia was carried out by approved methods. Echocardiography was performed in longitudinal and transverse axes using the M-mode and color/pulse Doppler at the level of the left ventricular outflow tract. The heart function was evaluated from the left parasternal long axis with the beams directed 25° cranially. The focus area was set at 10–15 mm. 2D images of the liver, kidneys, chest, and abdominal cavities were obtained. Doppler techniques were applied for blood flow analysis in the portal vein, inferior vena cava, and renal vessels with a 60° angle of insonation. The rat organs were characterized by US using similar criteria and patterns as those used in humans according to the suggested model [8,13,34]. We considered the US criteria of CHF as follows: liver enlargement, expanding of inferior vena cava, decrease of ejection fraction, and presence of ascites and hydrothorax.

Two invasive interventions (pleural cavity) for drug testing were used under US guidance. Fine needles, 29–31 G, were used. US was performed three times: on the same day and on the first and second day after the administration, in order to verify the CHF regression. Percutaneous intervention performed at adequate visualization of a target was carried out in the transverse axis (out of plane) following the general principles of interventional sonography.

### The second stage (comparative assessment the cardioprotective efficacy)

For comparative assessment, the cardioprotective efficacy of injection gold nanoparticle, gold nanoparticle conjugated with Simdax and Simdax only intrapleurally and intravenously. The laboratory Wistar rats (*N* = 48) were used for this stage within 2 weeks after intravenous injection of doxorubicin Sigma solution in cumulative dose of 12.0 mg/kg to simulate the heart failure according to experimentally established scheme. Animals showed an advanced heart failure that was caused by intravenously introduced doxorubicin (cumulative dose 12.0 mg/kg), as described in stages 1 and 2 of the study. Afterward, at the 14th day, animals with severe heart failure were assessed by ultrasound and were administered the following agents: (1) gold nanoparticles (2) Simdax (levosimendan), an inotropic and vasodilator agent, (3) gold nanoparticles and Simdax conjugate. Three kinds of solutions were administered in two different routes: into the pleural cavity (intrapleural injection under US guidance) or intravenously.

We formed six groups with eight rats in each (Table 2): the first three groups of animals received levosimendan (Simdax, Finland), gold nanoparticles, and conjugate (Simdax, gold), respectively, into pleural cavities in a dose of 0.06 ml per animal. The fourth, fifth, and sixth groups of animals received Simdax, gold nanoparticles, and conjugate (Simdax, gold), respectively, intravenously in same dose. We considered hydrothorax as the most representative sign for effective dynamic assessment of heart failure regression. The seventh group of animals (controls) received saline. Animals were observed until *natural death*, and afterward dissected and studied with light optical microscopy, laser correlation spectroscopy, and scanning electron microscopy (SEM).

**Table 2:** Comparative assessment of cardioprotective efficacy of AuNP injection, AuNPs-Simdax, and Simdax only intrapleurally and intravenously

**Group (*n *= 8)**	**Medication, route of injection**	**Hydrothorax**		
		**First examination**	**Second examination**	**Third examination**
1	AuNPs intrapleural	Right 1.8 ± 0.11 mm; left 2.1 ± 0.13 mm	No hydrothorax	No hydrothorax
2	AuNPs intravenous	Right 1.5 ± 0.09 mm; left 3 ± 0.16 mm	Right 0.5 ± 0.1 mm; left no hydrothorax	No hydrothorax
3	AuNPs-Simdax intrapleural	Right 0; left 1.8 ± 0.15 mm	No Hydrothorax	No hydrothorax
4	AuNPs-Simdax intravenous	right 0; left 0	Right no hydrothorax; left 1 ± 0.2 mm	No hydrothorax
5	Simdax intrapleural	Right 3 ± 0.12 mm; left 4.6 ± 0.26 mm	No hydrothorax	No hydrothorax
6	Simdax intravenous	Right 4 ± 0.16 mm; left 5.5 ± 0.21 mm	Right no hydrothorax; left 1.4 ± 0.21 mm	No hydrothorax
7	Controls	Hydrothorax: right 6 ± 0.46 mm; left 7 ± 0.35 mm	Severe ascites, hydrothorax, liver enlargement	Decrease of liver, nephropathy, severe ascites, hydrothorax, pericardial effusion

### The third stage (sonoporation cardioprotective efficacy)

Consequently, we performed the interventional procedures under US guidance for four rats after modeling of heart failure (cumulative dose of doxorubicin ‘Sigma’ at 3.04 mg per animal or 15 mg/kg): two rats were injected with AuNPs into the pleural cavity with minimal insonation, and the other two were injected with AuNPs into the pleural cavity, which is connected to the pericardial cavity. After identification of spread near the heart muscle, the targeted locus of myocardium in depth of 1 cm during 180 s was insonated by 130 Db ultrasound using multifrequency 3–8 MHz probe.

We performed a dynamic assessment of heart failure regression measuring hydrothorax *in vivo*. After necropsy on the seventh day, we performed pathomorphological and histological analyses of experimental study.

Light optical microscopy of myocardium samples were stained with hematoxylin-eosin. Semi-fine sections were stained with methylene blue. Using the light microscope Docuval Carl Zeiss (Jena, Oberkochen, Germany), images of the investigated object were captured.

For SEM, pieces of myocardium were fixed in 2.5% glutaraldehyde solution, then co-fixated by 1% solution osmic acid. Further dehydration and pouring in resin (Epone 812 or a mixture of the eponym aralditom) was carried out by the conventional method. Cutting blocks was held by ultratome LKB-III (Sweden) using glass knives on the device Knife Maker 7801B (LKB, Sweden). Ultrathin sections of thickness 50–60 nm with 2% solution of uranila acetate and lead citrate were used thus achieved the best contrasting sections. Some samples of infarction rats were examined without staining. Ultrathin sections were examined with an electron microscope TEM - 125 K (Ukraine).

Visualization of gold nanoparticles and their interaction with cells was performed using laser correlation spectroscopy (Zetasizer-3, Malvern Instruments Ltd, UK), transmission electron microscopy (JEM-1230, JEOL, Tokyo, Japan), scanning electron microscopy (JSM-35C JEOL, Japan), and confocal microscopy (LSM510 META, Carl Zeiss, Oberkochen, Germany).

Evaluation of activity of the drug was carried out by comparing the morphological changes of internal organs according to US and echocardiography and mortality of animals in research groups.

US criteria were documented according to the following parameters: the size of the liver, liver parenchyma density, large diameter vein circulation (inferior vena cava), hepatic veins, renal veins, the renal portal system blood flow, the presence of ascites and hydrothorax, etc. The system hemodynamics was complexly evaluated assessing the target internal organs.

All animals were kept in a vivarium (plastic cages, in separate rooms) at constant temperature (20–25°C), and inversed cycle of light–dark (12–12 h), with free access to food and water. Condition was observed daily for 21 days. The animals were fed by standard granulated food, according to the guidelines on Pets in the vivarium (Kyiv, 1976), and approved by the Ministry of Health of Ukraine.

The medical ethics commissions of Zabolotny Institute of Microbiology and Virology of National Academy of Sciences of Ukraine approved the study.

Statistical method of Mann–Whitney U test was applied to perform comparison between groups.

## Results

### Synthesis of gold nanoparticles of different sizes and their physico-chemical characteristics (performed in Ovcharenko Institute of Biocolloidal Chemistry of National Academy of Sciences of Ukraine)

The concentration of obtained gold nanoparticles was 38.6 μg/ml by metal, respectively, for each size. Samples of aqueous dispersions of gold nanoparticles of different sizes, synthesized using the condensation method of Davis, are presented in Figure 1.

### Interaction of gold nanoparticles with eukaryotic cells

**Figure 2:**
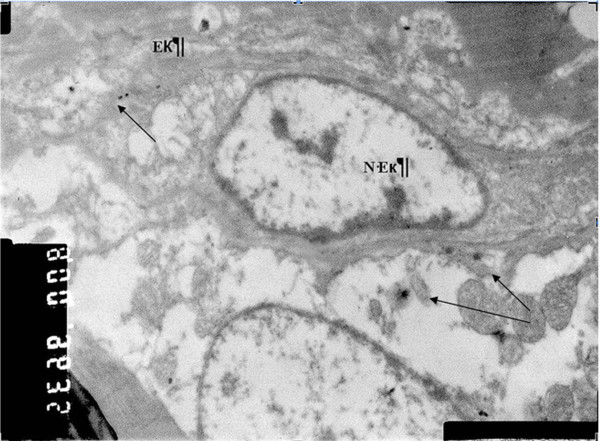
SEM image of cell line U937 (×3600).

To carry this out, cell line U937 (human leukemic monocyte lymphoma) was used; electron microscopy images are presented in Figure 2. These cells demonstrated their ability to actively accumulate gold nanoparticles, which were studied in all sizes on the surface and inside cells.

**Figure 3:**
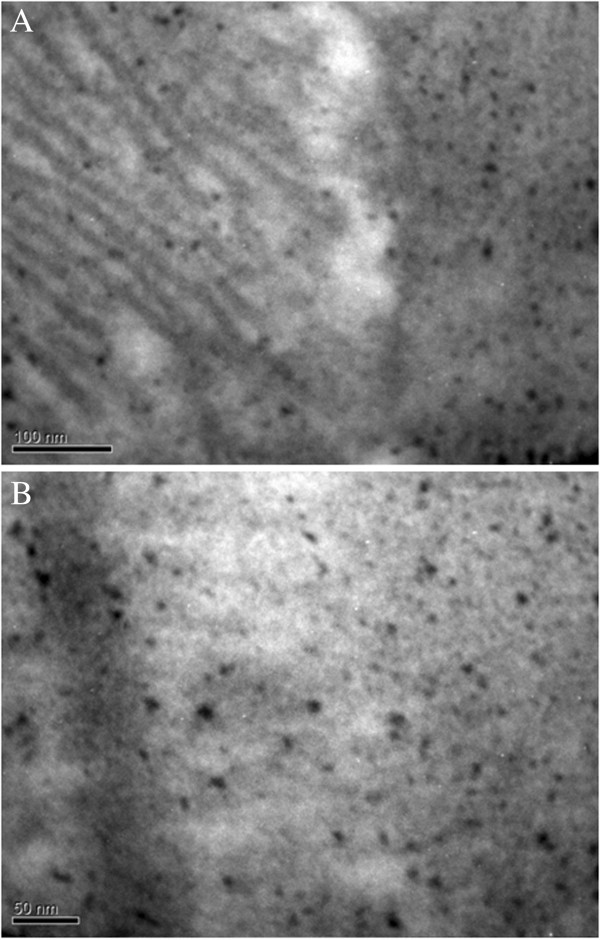
**Confocal microscopy image cell line U937 (final concentration of 106 cells/ml) after incubation for 3 min.** With gold nanoparticles of 20 nm **(A)** and 30 nm **(B)** in a final concentration of 12.7 μg/ml by metal. Scanning on the *z*-axis at intervals of 1 mm.

The most efficient cells accumulate gold nanoparticles of 20 and 30 nm. Figure 3 presents confocal microscopy images of layered scan cell line U937 after their interaction with gold nanoparticles, confirming the high level of accumulation, as evidenced by changes in the intensity of color in the pictures (from red (maximum) to blue (least)).

**Figure 4:**
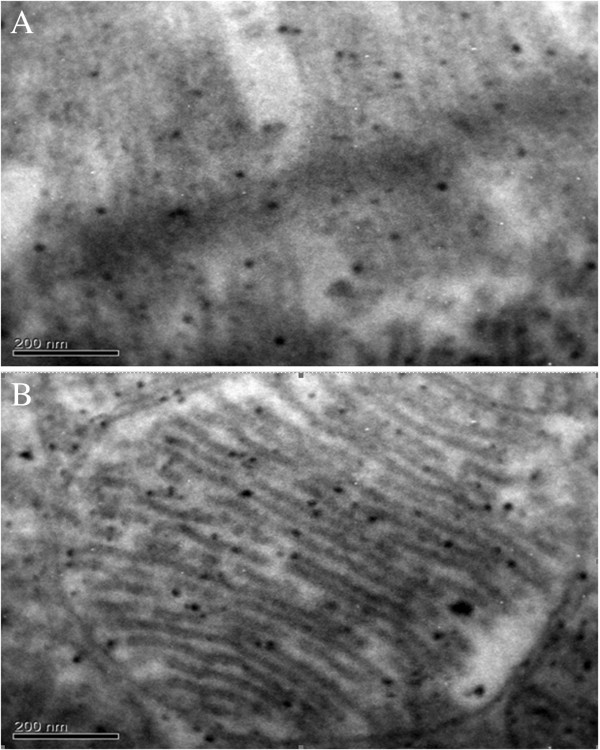
**Interaction of AuNPs measuring 10 nm with cell line U937 (final concentration 12.7 μg/ml by metal). (A)** Concentration curve of binding (*M* ± *m*; *n* = 5, *P* < 0.05), **(B)** Confocal SEM image.

**Figure 5:**
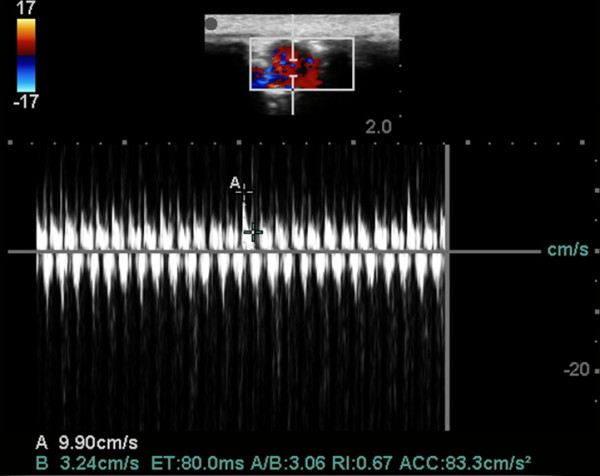
**Interaction of AuNPs of 20 nm with cell line U937 (final concentration 12.7 μg/ml by metal). (A)** Concentration curve binding (*M* ± *m*; *n* = 5, *P* < 0.05), **(B)** Confocal SEM image.

Experimentally, the interaction between cell line U937 with gold nanoparticles characterized by pronounced concentration dependence in the concentration range of 101–106 cells/ml for all studied nanoparticle sizes. Thus, the maximum binding concentration of nanoparticles of sizes 10 and 20 nm is 103 cells/ml (Figures 4 and 5).

It should be noted that the curve of binding for 10 nm gold nanoparticles measuring passes through a minimum at a concentration of 105 cells cells/ml, which may be due to reduction of active cell surface as a result of flocculation. This further increases the concentration of cells and leads to increased accumulation of gold nanoparticles to the maximum level by increasing the active surface area. That is, in the cells with nanoparticles, 10-nm concentration of 105 cells /ml is critical for cell-cell interactions.

**Figure 6:**
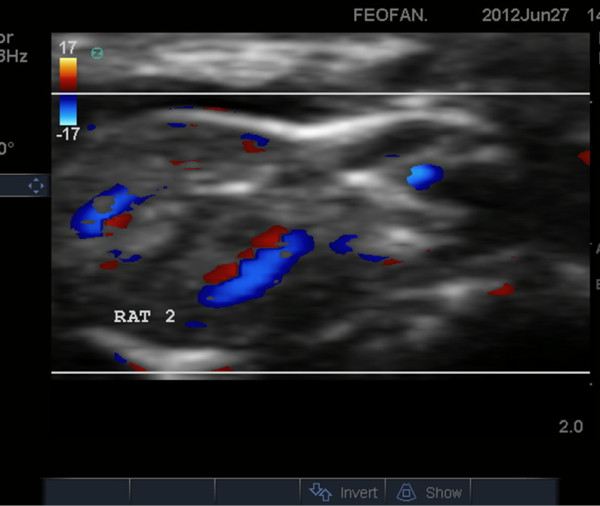
**Interaction of 30-nm gold nanoparticles with cell line U937 (final concentration 12.7 μg/ml by metal). (A)** Concentration curve of binding (*M* ± *m*; *n* = 5, *P* < 0.05). **(B)** Confocal SEM image.

Concentration curve of binding 30-nm nanoparticles differs from the previous two extended maximum, which is in the concentration range of 103–105 cells/ml (Figure 6).

**Figure 7:**
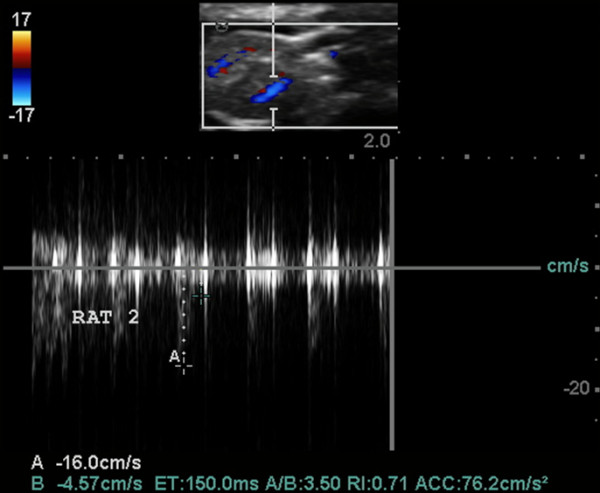
**Interaction of 45-nm gold nanoparticles with cell line U937 (final concentration 2.7 mg/ml by metal). (A)** Concentration curve of binding (*M* ± *m*; *n* = 5, *P* < 0.05). **(B)** Confocal SEM image.

Regarding the 45-nm gold nanoparticles, the binding curve almost reaches a plateau in the concentration range of 103–106 cells/ml and is characterized by a weakly pronounced maximum at a concentration of 104 cells/ml (Figure 7).

Changes in interaction efficiency of cell line U937 gold nanoparticles are most likely related to low concentrations of cells (from toxic effects of gold nanoparticles in the field of high concentrations of cells, a decrease in the area of the active surface contact of cells with nanoparticles due to the overwhelming intercellular interactions, that is, there are certain optimum ratio of cells and gold nanoparticles that determine the efficiency of binding.

**Figure 8:**
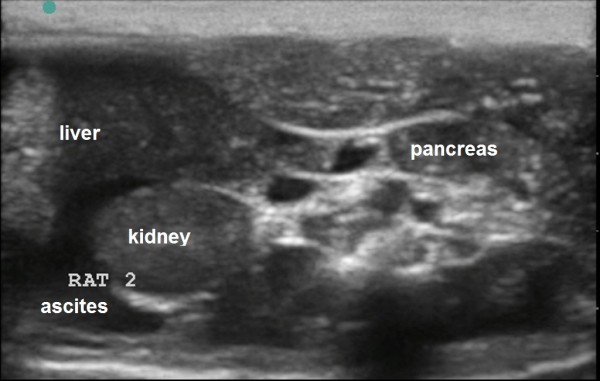
**Kinetics of binding cell line U937 gold nanoparticles.** With sizes 1–10, 2–20, 3–30, and 4–45 nm (*M* ± *m*; *n* = 5, *P* < 0.05). The final concentration (106 cells/ml) with gold nanoparticles (12.7 μg/ml by metal).

In terms of a single-cell concentration, optimum interaction of gold nanoparticles with cell line U937 is in the range of approximately 0.1–1.0 ng metal nanoparticles for all studied sizes. Kinetics of this process shows that the process is characterized by its high speed; its maximum binding of gold nanoparticles by cells is achieved within 3–5 min (Figure 8).

It should be noted that for 10-nm nanoparticles, after reaching the maximum level of accumulation, a reverse effect was observed (*reducing binding time*). This may be due to the ability of cells to the active ‘release’ nanoparticles of this size (Figure 8, curve 1).

**Figure 9:**
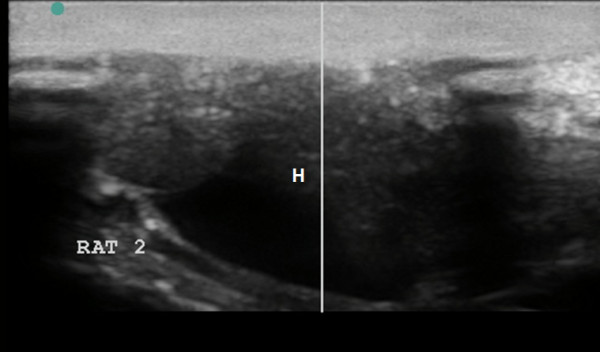
**SEM image of the intracellular localization of gold nanoparticles in vacuoles cell line U937.** Sizes of 20 nm **(A)** and 10 nm **(B)**. Cells at a final concentration of 106 cells/ml were incubated in the FSB buffer for 3–5 min with gold nanoparticles (final concentration of 12.7 μg/ml by metal).

After entry into the cell, gold nanoparticles have a different localization, depending on the size. Judging from electron microscopic analysis, we can speak about preferential accumulation of gold nanoparticles measuring 10 and 20 nm in the vacuoles (Figure 9).

**Figure 10:**
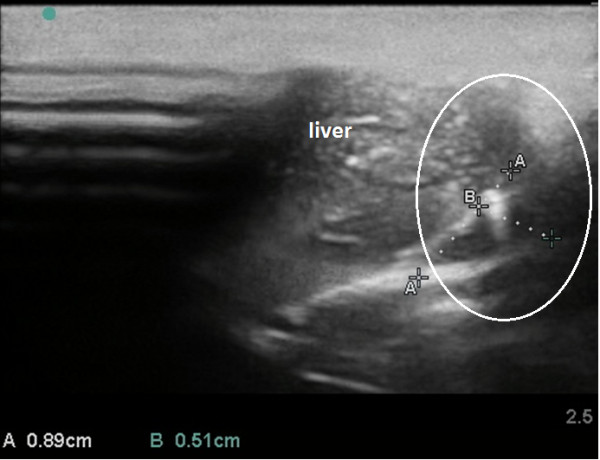
**SEM of the intracellular localization of 30-nm gold nanoparticles in lysosomes cell line U937.** Cells at a final concentration of 106 cells/ml were incubated in the FSB buffer for 3–5 min with gold nanoparticles (final concentration of 12.7 µg/ml by metal). Gold nanoparticles are observed on the lysosomes surface **(A)** and penetrated into the membrane protrusions **(B)**.

As can be seen in places likely hit, nanoparticles in vacuoles observed protrusion of their membranes, which may indicate the penetration of gold nanoparticle average size 10 and 20 nm inside vacuoles. For gold nanoparticles of 30 and 45 nm, their vast accumulation of lysosomes were observed (Figure 10).

### Effects of gold nanoparticles on the enzymatic activity of cell line U937

**Figure 11:**
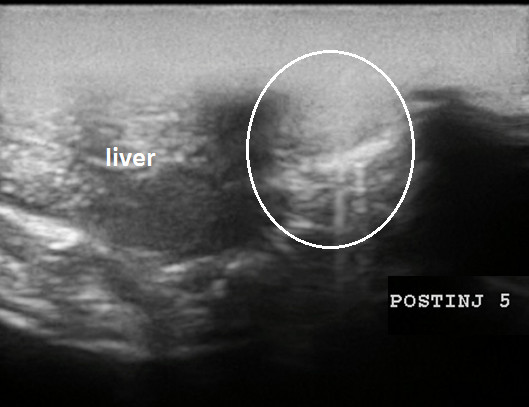
**Change in value of Na**^**+**^**, K**^**+**^**-ATP-ase activity (A/A**_**0**_**%) of membrane fraction from cell line U937 under AuNP influence.** Sizes of 1–10, 2–20, 3–30, and 4–45 nm. (*M* ± *m*; *n* = 5, *P* < 0.05 relative to control A_0_). For 100% (control) adopted the value Na^+^, K^+^-ATP-ase activity in the absence of gold nanoparticles. Gold nanoparticles in the environment made fiber membrane fraction (150–200 mg) and the mixture was incubated for 3 min. Incubation medium for determination of enzyme activity (volume 1 ml) is 50 mM Tris–HCl, 5 mM MgCl_2_, 100 mM NaCl, 20 mM KCl, 3 mM ATP (pH = 7.5). Incubation period is 10 min, and temperature is 37°C. The amount of membrane protein is 15–20 mg.

We found the significant changes in the presence of gold nanoparticles in the incubation medium size observed in Na^+^, K^+^-ATP-ase activity of the membrane fraction of cells (Figure 11, curves 1–4).

Thus, 10 nm gold nanoparticles in the concentration range of 0.11–1.1 mg/ml inhibit enzyme activity by 70% compared with the control (curve 1). Inhibition of Na^+^, K^+^-ATP-ase activity in the presence of gold nanoparticles of 20 nm is 20% (curve 2). Gold nanoparticles of 30 nm in the concentration range of 0.11–1.10 mg/ml stimulated the Na^+^, K^+^-ATP-ase activity; the value index, with increasing concentration of nanoparticles, increases and reaches 30%–40% in the concentration range of 0.28–1.10 mg/ml (curve 3). Under the influence of gold nanoparticles of 45 nm, an increase in Na^+^, K^+^-ATP-ase activity by 20%–40% was observed (curve 4). Thus, within the concentrations, 0.11–0.28 mg/ml for stimulation of metal is 20%. In the concentration range of 0.28–0.55 mg/ml by metal, enzymatic activity increases from 20% to 40% and averaged at 40% for the concentration range of 0.55–1.10 mg/ml by metal. Mg^2+^-ATP-ase activity of membrane fraction of cell line U937 under the influence of gold nanoparticles of average sizes 10, 20, 30, and 45 nm were not significantly changed. Features of gold nanoparticles on Na^+^, K^+^-ATP-ase activity membrane fraction cell line U937 can be caused by the interaction of nanoparticles of a certain size with SH-groups of the enzyme molecule responsible for its conformational state.

### The second stage

In all animals of the six groups, after the third day of post-medication injection, no ascites and no liver enlargement were registered (compared to controls *P* < 0.001). The linear cranio-caudal measurements of fluid level in the pleural cavities were as follows: for gold nanoparticles intrapleural right 1.8 ± 0.11 mm, left 2.1 ± 0.13 mm, compared to hydrothorax in controls right 6 ± 0.46 mm, left 7 ± 0.35 mm. Conjugate injection showed significantly higher hydrothorax reduction than that using Simdax injection only (*P* < 0.01); gold nanoparticle injection showed significantly higher than that using Simdax injection (*P* < 0.05). Gold nanoparticles and conjugate showed no significant difference in rat recovery.

**Figure 12:**
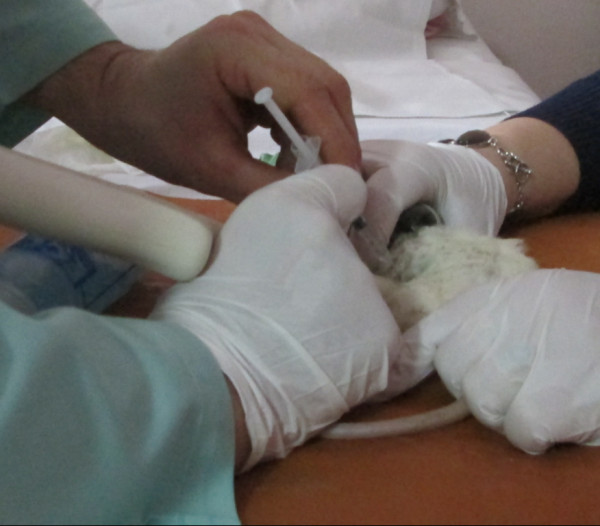
**Rat ultrasound visualizations. (A)** US survey. **(B)** echocardiography. **(C)** Doppler assessment of LV outflow tract [34].

**Figure 13:**
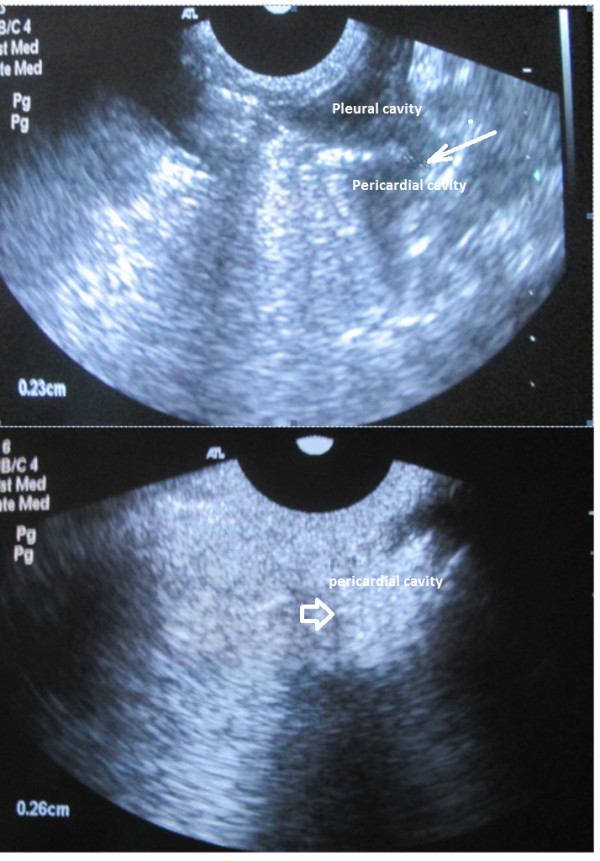
**Liver sonogram. (A)** Inferior vena cava (IVC) expanded to 4.5 mm, **(B)** dilated hepatic veins (HV), indirect sign of venous congestion in large circulation, and **(C)** mild ascites in rat. Near liver strip of liquid is revealed in **(A)**, an indirect sign of venous congestion in large circulation.

**Figure 14:**
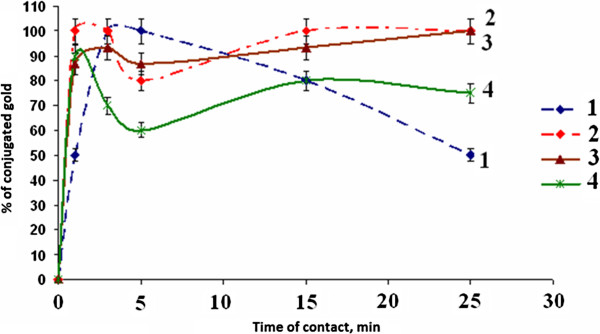
**US scans. (A)** an indirect sign of kidney venous congestion. IR in renal segmental artery is 0.67 (normal), **(B)** sonogram demonstrates kidney blood perfusion and ascites, **(C)** sign of nephropathy, IR in renal segmental artery is 0.71.

**Figure 15:**
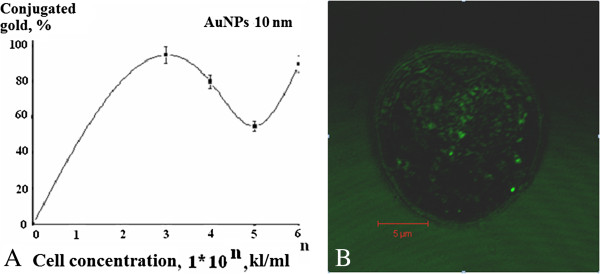
**US scans severe venous congestion. (A)** Severe ascites on cross-section abdominal scan. **(B)** Severe hydrothorax (H) on sagittal thoracic scan.

**Figure 16:**
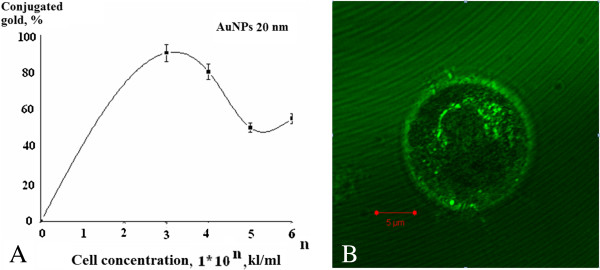
**Pleural effusion assessment in rat (sagittal US scans). (A)** Free fluid revealed in the pleural cavity. **(B)** No hydrothorax revealed 3 days after intrapleural injection of gold nanoparticle.

**Figure 17:**
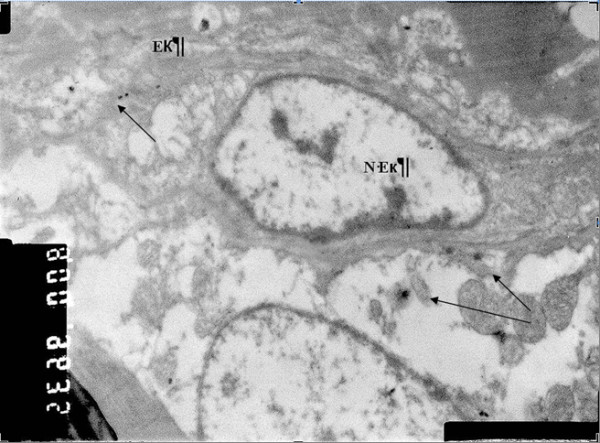
**Injection into pleural cavity. (A)** Procedure view and **(B)** spreading to the pericardial cavity through the pore (thin arrow), liquid in the pericardial cavity (arrow) [34].

We obtained sufficient ultrasound visualization of the affected organs (Figures 12, 13, 14, 15, 16, and 17); the evolution of symptoms in chosen groups with polar outcomes (controls and intrapleural conjugate) is presented in Tables 3 and 4.

**Table 3:** Heart failure rats, which received intrapleural conjugate of gold nanoparticles and Simdax, n = 7

**Animal**	**First examination**	**Second examination**	**Third examination**
Rat 1	Mild ascites (Figure 3), IVC expanded (Figure 4), liver enlargement	Mild ascites, liver enlargement	No symptoms
Rat 2	Mild ascites, IVC expanded, liver enlargement, mild hydrothorax	Mild ascites, IVC expanded, liver enlargement	Reduction of pericardial and pericardial effusion
Rat 3	IVC expanded, liver enlargement	Mild ascites, mild hydrothorax (Figure 5A), IVC expanded, liver enlargement	Reduction of pericardial and pericardial effusion
Rat 4	IVC expanded, liver enlargement	IVC expanded, liver enlargement	IVC expanded
Rat 5	Mild ascites IVC expanded, liver enlargement	Mild ascites, hydrothorax, pericaridial effusion	Reduction of pericardial and pericardial effusion
Rat 6	Mild ascites IVC expanded, liver enlargement	Ascites, hydrothorax, pericaridial effusion	Reduction of pericardial and pericardial effusion
Rat 7	IVC expanded, liver enlargement	Mild ascites, hydrothorax, IVC expanded, liver enlargement	IVC expanded, liver enlargement

**Table 4:** **Rat controls (heart failure rats), *n *= 7**

**Animal**	**First examination**	**Second examination**	**Third examination**
Rat 1	Ascites, hydrothorax, liver enlargement	Decrease of ejection fraction, severe ascites, hydrothorax, pericardial effusion	Decrease of liver, nephropathy, severe ascites, hydrothorax, pericardial effusion
Rat 2	Ascites, hydrothorax, liver enlargement	Severe ascites, hydrothorax, pericardial effusion.	Decrease of liver, nephropathy (Figure 5), severe ascites (Figure 6), hydrothorax, pericardial effusion
Rat 3	Ascites, liver enlargement	Severe ascites, hydrothorax, liver enlargement	Decrease of ejection fraction, severe ascites, hydrothorax, pericardial effusion
Rat 4	Ascites, hydrothorax, liver enlargement	Severe ascites, hydrothorax, pericardial effusion.	Decrease of liver, nephropathy, severe ascites, hydrothorax, pericardial effusion
Rat 5	Ascites, hydrothorax, liver enlargement	Severe ascites, hydrothorax, liver enlargement, pericardial effusion	Decrease of ejection fraction, severe ascites, hydrothorax, pericardial effusion
Rat 6	Ascites, hydrothorax, liver enlargement	Severe ascites, hydrothorax, liver enlargement	Decrease of liver, nephropathy, severe ascites, hydrothorax, pericardial effusion
Rat 7	Ascites, hydrothorax, liver enlargement	Severe ascites, hydrothorax, liver enlargement, pericardial effusion	Decrease of liver, nephropathy, severe ascites, hydrothorax, pericardial effusion

After the third examination post-treatment, hydrothorax remained the only US symptom for observation.

In all animals of the six groups after the third day after medication injection, physical conditions were observed as good. No ascites and no liver enlargement were registered in all animals of first six groups compared to signs of heart failure in controls (*P* < 0.001). The linear cranio-caudal measurements of fluid level were as follows: for gold nanoparticles, intrapleural right is 1.8 ± 0.11 mm and left is 2.1 ± 0.13 mm compared to hydrothorax in controls wherein right is 6 ± 0.46 mm and left is 7 ± 0.35 mm. Conjugate injection showed significantly higher hydrothorax reduction than that using Simdax injection only (*P* < 0.01). Gold nanoparticle injection showed significantly higher results than that using Simdax injection (*P* < 0.05). Gold nanoparticles and conjugate showed no significant difference in rat recovery. The study of route of injection indicated that intrapleural injection showed better and faster results in reducing hydrothorax on the second exam in all groups (*P* < 0.05).

Mean life continuity was as follows:

1) for gold nanoparticles, intravenous injection had 5.4 months while intrapleural injection had 6.2 months;

2) for Simdax, intravenous injection had 3 months while intrapleural injection had 3.4 months; and

3) for gold nanoparticle and Simdax conjugate, intravenous injection had 6.8 months while intrapleural injection had 6.3 months.

### Difference was significant between Simdax vs nanogold (*P* < 0.05) and Simdax vs conjugate (*P* < 0.05)

Table 4 depicts cardioprotective effects of all three substances using the two routes.

Structural changes in the myocardium, defined by light optical morphological study of heart tissue samples of rats with doxorubicin-induced heart failure, revealed emergence of larger lesions with contraction-damaged appearance of small foci vacuole degeneration and lysis of myofibrils. The results of injuries after modeling CHF were necrobiosis and necrosis of cardiomyocytes (CMC), which was accompanied by focal proliferation of connective tissues around dead CMC and the formation of small scars on the background of the diffuse proliferation of connective tissues and hypertrophy of individual CMC. These findings were most expressed in controls (Figures 12, 13, 14, and 15). After injection, 30-nm gold nanoparticles were found to be accumulated in the endothelial cells of infarcted arterioles and capillaries. Necrobiosis and scarring was significantly decreased after all treatments.

### The third stage

**Figure 18:**
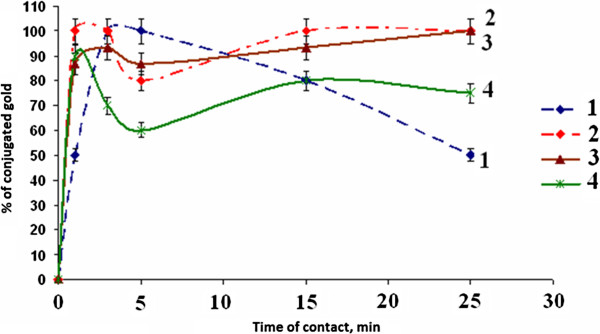
**Light optical microscopy, painted by hematoxylin-eosin (×200). (A)** CHF modeling in rat: CMC contraction damage, necrosis, nodular proliferation of connective tissue cells, and necrosis of nearby vessels; **(B)** after conjugate injection: slight nuclei hypertrophy of CMC, little amount of connective tissue.

In the sonoporation of rats, CHF symptoms and hydrothorax were totally gone on the first day after injection vs reduction on the third day (Figure 16). Figure 17 demonstrates the spreading of medication from the pleural to the pericardial cavity to obtain contact with myocardium to targeted sonoporation performance. Sonoporation is able to enhance gold nanoparticle delivery to myocardial cells *in vivo*, and electron microscopy of the myocardium of experimental rats showed that ultrasound enhances AuNP transfer into the cell and mytochondria which were highly localized, and that result was superior to that of controls (*P* < 0.01 for both). US changes after CHF modeling and after conjugate injection are presented in Figure 18.

**Figure 19:**
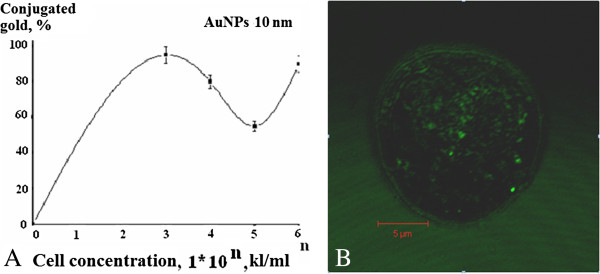
**The Ca**^**2+**^**, Mg**^**2+**^**-ATP-ase activity in myofibrils of heart for Simdax, AuNPs, and conjugate.**

The obtained data showed that the effect of 20-nm gold nanoparticle average (initial drug concentration 193 mg/ml by metal) on the value of myofibrils Ca^2+^, Mg^2+^-ATP-ase activity marked the inhibition of enzyme activity in the whole investigated concentration range of nanoparticles on average (20%–30%) and under the concentration of nanoparticles in the incubation medium 0.051 mg/ml by metal (37%) compared with control (Figure 19).

AuNPs-Simdax conjugate unlike Simdax normalized Ca^2+^, Mg^2+^-ATP-ase activity of heart myofibrils (Figure 19). We assume that this will help avoid potential side effects of Simdax including tachycardia or development of congestive heart failure.

**Figure 20:**
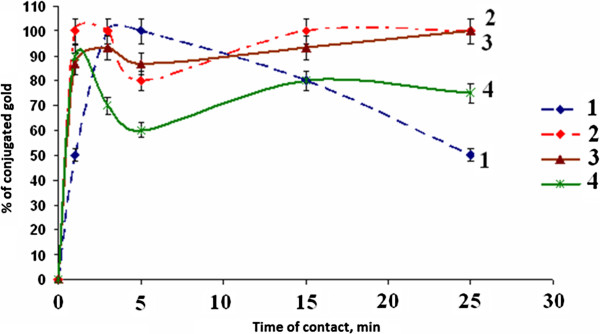
**SEM (×16,000).** Accumulation of 30-nm gold nanoparticles in arteriolar endothelium of left ventricular myocardium after intravenous injection. In the cytoplasm of endothelial cells, electron-dense inclusion of gold nanoparticles (indicated by arrows) was revealed. (EK) endothelial cells, (N-Ek) nucleus of endothelial cells, (arrows) NPs.

**Figure 21:**
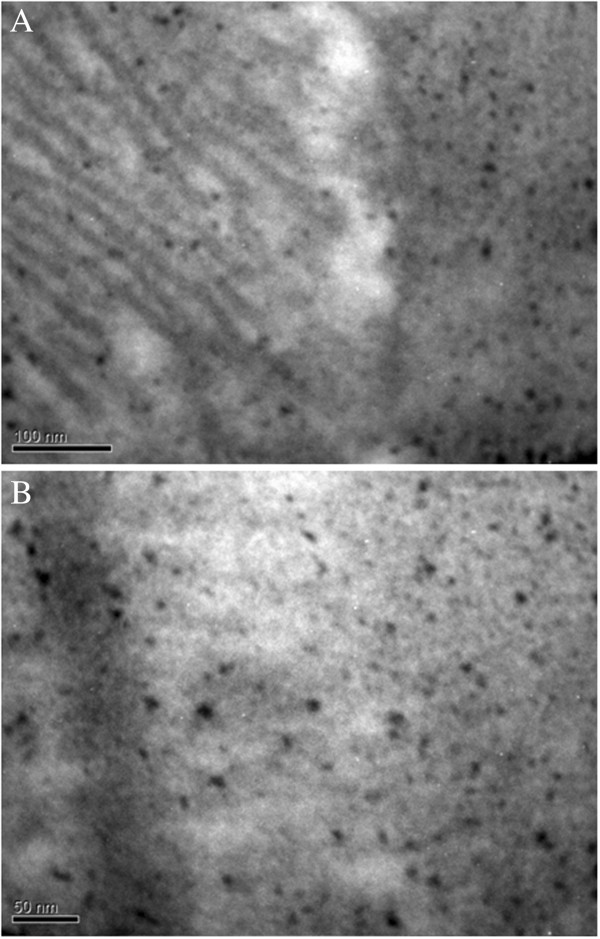
**SEM (×250,000).** Cardiomyocytes after NP injection to intact rats. **(A)** Myofibrils. **(B)** Mitochondria.

**Figure 22:**
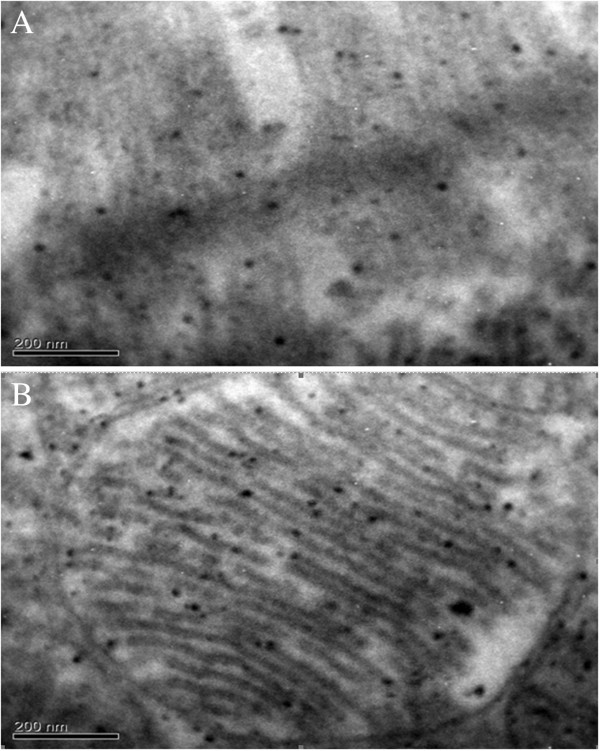
**SEM (×250,000).** Inclusion of NPs in **(A)** myofibrils and **(B)** mitohondrias. CMC after sonoporation (larger amount of particles is observed).

Electron microscopic examination on AuNPs-Simdax conjugate samples revealed tropism of myofibrils and mitochondria of CMC for intravenous and intrapericardial injection. After 1 h, conjugate was detected in a small number of inclusions in the mitochondria myofibrils and CMC (Figure 20). Figures 21 and 22 demonstrate the difference of the accumulation of NPs in the myofibrils of CMC and mitochondria between regular injection and sonoporation groups. The conjugate was detected in a larger number of inclusions after myocardium insonation.

## Endnotes

### Findings of biosafety and biocompatibility tests of gold nanoparticles and conjugate with Simdax

• Genotoxicity: eukaryotic cells under the influence of gold nanoparticles and AuNPs-Simdax conjugate was at a negative control and did not exceed 0.3%;

• no mutagenic effect was found: gold nanoparticles and conjugate AuNPs-Simdax did not cause micronuclei in cell formation in laboratory animals (allows to predict the absence of such influence on the human body);

• no influence on intestinal microflora in intact rats (allow to predict the absence of such influence on the human body);

• no impact on immunoreactivity *in vivo* was registered (cytokine production, phagocytes, indicators of cellular immunity); and

• most gold nanoparticles of 30 nm are biologically safe and biocompatible *in vitro* and *in vivo*; based on 30-nm gold nanoparticles, AuNPs-Simdax conjugate was found the most biologically safe according to cytotoxicity, genotoxicity, and immunoreactivity.

### Findings of assessment of cardioprotective properties and route of delivery of gold nanoparticles

• Ultrasonography is an effective modality for *in vivo* monitoring the condition of rat organs targeted for experiment for the study of cardiovascular function.

• The results of doxorubicin-induced injuries in myocardium on rat model are necrobiosis and necrosis of CMC, which was accompanied by focal proliferation of connective tissue around dead CMC and the formation of small scars on the background of diffuse proliferation of connective tissue and CMC hypertrophy.

• Intravenous injection of 30-nm gold nanoparticles were found to be accumulated in the endothelial cells of infarcted arterioles and capillaries. Necrobiosis and scarring was significantly decreased after all treatments.

• AuNPs-Simdax conjugate showed a positive effect on the cardiac contractile ability (level of energy costs) of conditionally healthy animals. Conjugate injection showed significantly higher hydrothorax reduction than injection of Simdax only (*P* < 0.01); gold nanoparticle injection showed significantly higher than injection of Simdax (*P* < 0.05).

• Gold nanoparticles and conjugate showed no significant difference in rat recovery.

• In the study of the route of injection, intrapleural injection showed better and faster results in reducing hydrothorax on the second exam in all groups (*P* < 0.05).

• Preliminary results indicate higher mortality after Simdax administration and the longest survival after conjugate administration.

• Intrapleural (local delivery) route is preferred over intravenous (systemic) according to all tested parameters.

### Theranostic potential

Sonoporation showed to enhance gold nanoparticle delivery to myocardial cells *in vivo*. Thus, after assessing relevant parameters as AuNPs demonstrated not only significant drug delivery properties but also discover strong inotropic cardioprotective activity that led to significant life extension compared with one of the most effective existing inotropic agents, being biosafe and biocompatible.

## Discussion

Considering the results of this study and literature data demonstrating strong antioxidative properties, biosafety of gold nanoparticles are promising for advanced therapy development and innovative drug delivery stratification and personalization of treatments in novel disciplines [6] such as nanocardiology [77,78], nanoendocrinology [70], and nanoneurology [37,71].

### Nanocardiology

The rapid development of nanomedicine has not bypassed cardiovascular diseases. Although the publications in this sphere, with the use of nanomaterials, is still quite a bit few, there are already attempts to use the nanoparticles as vectors targeted delivery of cardioprotective drugs [43]. Nanoscale particles can be synthetically designed to potentially intervene in lipoprotein matrix retention and lipoprotein uptake in cells (processes central to atherosclerosis). Nanoengineered molecules called *nanolipoblockers* can be used to attack atherosclerotic plaques due to raised levels of low-density lipoproteins [79]. An experimental study in rats using injectable self-assembling peptide nanofiber bound to platelet-derived growth factor demonstrated sustained delivery to the myocardium resulting in decreased cardiomyocyte death and preserved systolic function after myocardial infarction [80]. In studies on rats, cell therapy with insulin-like growth factor 1 delivery by biotinylated nanofibers improved systolic function after experimental myocardial infarction [81]. As various mechanisms enabling cardiac regeneration are becoming elucidated, novel technologies using degradable microspheres for controlled release systems and self-assembling peptide nanofibers for cell and factor delivery were reported [82].

Cardiovascular diseases are strongly connected to immune response. Pathogenesis of cardiovascular diseases are associated with dysfunction of cytokine production. In most autoimmune diseases observed, stereotyped response in the form of a large subpopulation of activated Th1 lymphocytes [83], not rarely observed, decrease in the number of T-lymphocytes, impaired T helpers/suppressors ratio downward suppressor activity, weakening the response to the mitogens. In patients with autoimmune disease, often increased levels of proinflammatory cytokines (TNF-α, IL-1, IFN-γ) may result aberrant activation of the innate immune response [84]. During a persistent heart muscle damage, the exposure of the intracellular content to dead cells activates the innate immune response, such as the activation of Toll-like receptors (TLR). In the heart, TLR2 and TLR4 are perhaps involved in the host response to myocardial infarction [85]. The activation of TLR initiates the imbalance of TLR-induced cytokines.

We hypothesize that the AuNPs may affect the calcium channels and have an impact on the imbalance of cytokines.

Nanogold is known to impact on receptors and gene expression. AuNPs bind strongly to thiols and amines and thereby inhibit VEGF165-induced signalling [86]. Giljohann DA et al. described *gene regulation* with polyvalent RNA-gold nanoparticle conjugates (RNA-Au NPs) [87]. Patel PC et al. identified the pathway for DNA-AuNP entry in HeLa cells by a process involving receptor-mediated endocytosis, mediated by a class of pattern-recognition receptors [88,89].

### Nanoneurology

Kogan et al. reported the use of local heat delivered by metallic nanoparticles selectively attached to their target as a molecular surgery to safely remove toxic and clogging aggregates, particularly the amyloid beta protein involved in Alzheimer's disease, a neurodegenerative disease [37].

We hypothesize, that due to NP bioeffects against cellular oxidative stress, targeted teranostic treatment for neuromuscular diseases (myopathy, neuropathy, latent trigger points) may be applied in the near future after approval from evidentiary studies [77]. Combination with targeted biology therapies as growth factor of platelet rich plasma [78] gives new opportunities for neuromuscular disease management.

Nanoneurosurgery is a conceptual leap necessary for neuroscientists as well as neurosurgeons in developing and applying nanotechniques to neurosurgery at the nano level. According to Andrews [70], nanoscaffolds offer mechanical enhancement of neurorepair; carbon nanotube electrode arrays can provide nanolevel electrical and chemical enhancement. Even the traditional ‘cut-and-sew’ surgery is being taken down to the micron, if not nano, level for single-axon repair, and the technology can use capillaries to deliver therapeutics to virtually any portion of the nervous system with greater-than-pinpoint accuracy.

Future calls for upcoming PPPM-related studies with particular applications of AuNPs for therapeutic drug delivery properties in multifunctional nanomedical solutions related to genetics and cell biology are required in the following fields:

• Nanohepatology

• Nanonephrology

• Nanoallergology

• Nanogastroenterology

### Consolidation of the PPPM concept

#### ***Personalized medical approach***

Imaging/sonoporation combined with direct visualization of target tissues and optoacoustic phenomena to detect nanoparticles *in vivo* and its potential to be a contrast agent for US/MRI imaging is a significant opportunity for personalized theranostics.

#### ***Predictive medical approach***

Optoacoustic phenomenon is a relevant basis for contrast imaging with biomarker registration with high predictive value potential. Extensive application of sensor based on AuNPs allows us to think about developing novel technologies for minimally invasive diagnostic/treatment procedures.

#### ***Preventive medical approach***

Strong antioxidative effects potential to impact on cellular receptors and gene expression combined with high biosafety is a crucial challenge in anti-aging strategy. Further studies are necessary to clarify the molecular mechanisms of AuNP effects and its relevant dosage.

### Study limitation

Although this research was carefully prepared with sufficient number of observations, we are still aware of its limitations. First of all, the research was conducted as part of a large study, concerning the creation of a rat model for testing new medications based on nanoparticles and drug delivery with the assistance of US. That is why some parameters were not presented in this paper. Secondly, our study was methodologically limited by use of general, not special US equipment for precise assessment of the heart tissue. The opticoacoustic phenomenon was not applied to detect nanoparticles *in vivo*. As CHF occurs in elderly humans, age-related comparative studies on rat models have to be relevant.

### Future outlooks and recommendations

Further studies dedicated to the mechanism of the cardioprotective effects of gold nanoparticles for delivering drugs and testing on different animal heart failure models, especially with relation to the age of the animal, are required. Molecular mechanisms are still not clear, and further studies are required. Different approaches for drug delivery may be suggested and should be tested, based on the combination of expressions by different physical properties, e.g., sonoporation or colloid conjugation, liposomes, etc.

A study on interactions with other nanomaterials (e.g., cerium dioxide, carbon nanomaterials) and combination with other biological (gene, regenerative) therapies are recommended. After approval, agent safety development medications with future clinical testing should be initiated to implement theranostic approach for routine practice.

With the concluding points, we can formulate the following proposals (expert recommendations):

1. For the European Union (EU): create an international project to study gold nanoparticles for the development of nanoconstructions to treat patients with heart failure. Extend studies to nanoparticle application in neurodegenerative, heart, liver, and kidney diseases and muscle dystrophy, combining with biological therapies to achieve sustainable effects from theranostic approach.

2. For Ukraine: participate in project in partnership with EU to follow up experimental and clinical trials and involve related institutions and centers to the study.

## Conclusions

Based on the results, we have concluded the following:

1. We developed and implemented original protocols for colloid-chemical synthesis of spherical gold nanoparticles of discrete sizes 10, 20, 30, and 45 nm and designed and constructed AuNPs-Simdax conjugate, and both were found to be biologically safe (in cytotoxicity, genotoxicity, and immunoreactivity).

2. Most gold nanoparticles of 30 nm and its AuNPs-Simdax conjugate are biologically safe and biocompatible *in vitro* and *in vivo*.

3. Conjugate AuNPs-Simdax showed a positive effect on the cardiac contractile ability of conditionally healthy animals (have significant cardioprotective effects for doxorubicin-induced heart failure rats, higher than Simdax only).

4. Gold nanoparticle injection itself showed effects similar to the AuNPs-Simdax conjugate injection.

5. Intrapleural (local) delivery is more effective over intravenous (systemic) according to all tested parameters.

6. Sonoporation is able to enhance gold nanoparticle delivery to myocardial cells *in vivo*.

## Abbreviations

PPPM: Predictive preventive and personalized medicine; NPs: Nanoparticles; AuNPs: Gold nanoparticles; AuNPs-Simdax: Conjugate based on gold nanoparticles and Simdax; CHF: Congestive heart failure; US: Ultrasound; CMC: Cardiomyocytes; SEM: Scanning electron microscopy.

## Competing interests

The authors declare to have no competing interests.

## Authors' contributions

MYS did the organization and analysis of the study. RVB developed the model and did the ultrasound survey, literature search, analysis of the study, and article preparation. IMY, LLM, and TNO developed the model and performed the biosafety tests. UZR synthesized the AuNPs and prepared the Simdax conjugate and performed the biosafety tests. IMY performed the analysis and SEM tests. All authors read and approved the final manuscript.

## Authors' information

Professor MYS, Ph.D., D.Sci., is a corresponding member of the National Academy of Sciences of Ukraine and the director of the Inteferon Department of Zabolotny Institute of Microbiology and Virology, NAS of Ukraine, Kyiv, Ukraine. RVB, M.D., Ph.D., is a medical doctor in the Clinical Hospital ‘Pheophania’ of the State Affairs Department, National Representative of the European Association for Predictive, Preventive and Personalized Medicine (EPMA) in Ukraine. Professor IMY, M.D., D.Sci., is the director of the Scientific-Practical Centre of Pediatric Cardiology and Cardiac Health of Ukraine, Kyiv, Ukraine, and was a Minister of Health in Ukraine (2010–2011). Professor LLM, Ph.D., D.Sci. and TNO, Ph.D., a researcher, are members of the Inteferon Department of Zabolotny Institute of Microbiology and Virology, National Academy of Sciences of Ukraine group. Professor UZR, Ph.D., D.Sci., is the director of the Ovcharenko Institute of Biocolloidal Chemistry, National Academy of Sciences of Ukraine, Kyiv, Ukraine.
